# What Role Does the Endocannabinoid System Play in the Pathogenesis of Obesity?

**DOI:** 10.3390/nu13020373

**Published:** 2021-01-26

**Authors:** Piotr Schulz, Szymon Hryhorowicz, Anna Maria Rychter, Agnieszka Zawada, Ryszard Słomski, Agnieszka Dobrowolska, Iwona Krela-Kaźmierczak

**Affiliations:** 1 Department of Gastroenterology, Dietetics and Internal Diseases, Poznan University of Medical Sciences, 49 Przybyszewskiego Street, 60-355 Poznan, Poland; piotrek.schulz@wp.pl (P.S.); aga.zawada@gmail.com (A.Z.); agdob@ump.edu.pl (A.D.); krela@op.pl (I.K.-K.); 2 Department of Physiology, Poznan University of Medical Sciences, 6 Święcickiego Street, 60-781 Poznan, Poland; 3 Institute of Human Genetics, Polish Academy of Sciences, Strzeszynska 32, 60-479 Poznan, Poland; szymon.hryhorowicz@gmail.com (S.H.); slomski@up.poznan.pl (R.S.)

**Keywords:** endocannabinoid system, obesity, cannabinoid receptors, obesity pathogenesis, obesity genes

## Abstract

The endocannabinoid system (ECS) is an endogenous signaling system formed by specific receptors (cannabinoid type 1 and type 2 (CB_1_ and CB_2_)), their endogenous ligands (endocannabinoids), and enzymes involved in their synthesis and degradation. The ECS, centrally and peripherally, is involved in various physiological processes, including regulation of energy balance, promotion of metabolic process, food intake, weight gain, promotion of fat accumulation in adipocytes, and regulation of body homeostasis; thus, its overactivity may be related to obesity. In this review, we try to explain the role of the ECS and the impact of genetic factors on endocannabinoid system modulation in the pathogenesis of obesity, which is a global and civilizational problem affecting the entire world population regardless of age. We also emphasize that the search for potential new targets for health assessment, treatment, and the development of possible therapies in obesity is of great importance.

## 1. Introduction

According to the World Health Organization (WHO), obesity is defined by a body mass index (BMI) higher than or equal to 30 kg/m^2^. Although the BMI is not an ideal diagnostic tool, it is widely used in clinical practice as well as in assessing the prevalence of obesity worldwide. However, it should be noted that the BMI uses only height and body weight, which could be misleading when muscle tissue is overgrown [1]. Therefore, the waist-to-hip ratio (WHR) should be measured to assess the location of adipose tissue [2]. In recent years, more accurate techniques, such as bioelectrical impedance analysis (BIA) or gold-standard dual-energy X-ray absorptiometry (DXA), have become more common, allowing a more precise evaluation of adipose tissue [3]. 

**Figure 1:**
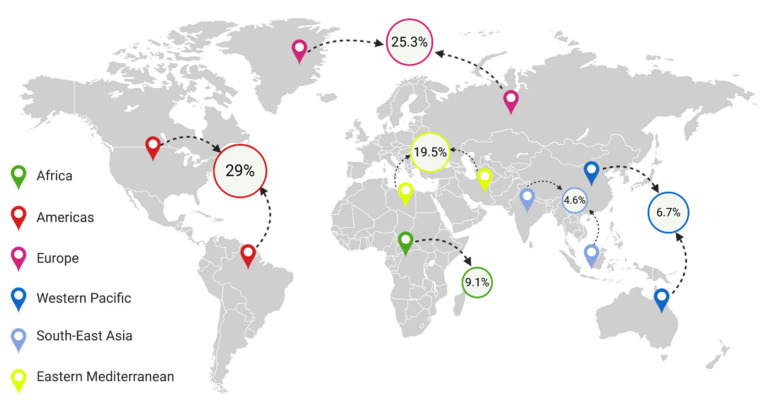
Percentage of people with obesity in different regions of the world [13,14,15,16,17].

Within a few decades, obesity has become a global problem. Currently, almost 2 billion individuals worldwide suffer from overweight, and over 650 million are obese (Figure 1) [4]. In Poland, 21.3% of adults suffered from obesity in 2016, with a slight predominance of men. On the other hand, in the U.S., 36.2% of the population, predominantly women, suffer from obesity [5]. In fact, the prevalence of obesity has almost tripled since 1975 [6], and it is estimated that by 2030, half of the world’s population will be overweight [7]. Currently, obesity, especially among children, is vastly increasing in developing countries, particularly in urban areas. Interestingly, the prevalence of obesity is more than 30% higher among children living in developing countries than among children living in developed countries [8]. It is essential to note that 75% of people with excessive body weight in childhood will suffer from overweight or obesity in adult life [9]. The pathogenesis of obesity is complex and has not been investigated thoroughly enough, although it is accepted that behavioral, genetic, and biological factors, including intestinal microbiota or even intrauterine growth, have been associated with the development of obesity [8,10,11]. 

There is evidence of a link between intestinal microbiota and obesity. It has been discovered that microbiological changes in the intestine constitute a risk factor for obesity among humans [12]. In addition, bariatric surgery partly improves excessive body weight, associated with intestinal dysbiosis, and the changes in the intestinal microbiota composition have been associated with positive results following the surgery, i.e., with weight loss or metabolism improvement [11].

However, the direct cause of obesity is excessive calorie intake. Nearly 3 million people in the world die of obesity-related comorbidities each year [5]. Furthermore, obesity is associated with a higher incidence of cardiovascular disease, including heart failure, which is the main cause of death globally. 

One of the elements responsible for the body’s nutrition and metabolic state is the food-intake-regulating system, involving numerous hormones, cytokines, and other transmitters, such as insulin, leptin, ghrelin, glucocorticosteroids, and endocannabinoids [18]. Food intake is regulated at both central and peripheral levels [19]. Central regulation is responsible for nutritional (pro-nutritional) behavior and the resulting feeling of pleasure, whereas peripheral regulation is based on the modification of lipogenesis, adipogenesis, glucose metabolism, and lipoprotein metabolism, in which the endocannabinoid system (ECS) plays a significant role [4,20].

The isolation of Δ9-tetrahydrocannabinol (Δ9-THC) in 1964 was a milestone in discovering the endocannabinoid system [21]. Studies on Δ9-THC led to the identification of the first cannabinoid receptor, i.e., cannabinoid receptor type 1 (CB_1_R) in the rat brain, which was classified as a G-protein-coupled receptor (GPCR) [22]. In the following years, CB_1_R was found in many species, including humans [23]. In 1993, a second cannabinoid receptor, cannabinoid receptor type 2 (CB_2_R), also classified as a GPCR, was discovered in spleen macrophages [24]. It is worth mentioning that other types of cannabinoid receptors, nonCB_1_ and nonCB_2_ orphan GPR55 and TRPV1 receptors, have been reported so far, which may explain the pathway that has not been fully understood [25,26,27]. However, to date, no genetic polymorphism changes in GPR55 and TRPV receptors have been identified in terms of their role in determining obesity. Only in vivo studies on the role of GPR55 in energy and glucose homeostasis have been performed in GPR55-/- mice, which revealed that GPR55 knockout, at least partially, increased adiposity and insulin resistance due to reduced physical activity [28]. A similar observation was confirmed for the TRPV receptor, where the authors evaluated the effect of capsaicin on the browning program in white adipose tissue (WAT) by the activation of TRPV1 channels to prevent diet-induced obesity in wild-type and TRPV1(-/-) mouse models. They successfully demonstrated that activation of TRPV1 channels by dietary capsaicin results in the browning of WAT, thus prevent obesity, which implies that TRPV could become a promising new target to combat obesity [29]. This fact was confirmed by Christie et al. in 2018, which indicates that TRPV may be involved in energy homeostasis and the control of food intake, appetite, and energy expenditure. This, in turn, strongly suggests that its dysregulation may be involved in the development of obesity. The mechanisms causing dysregulation have not been fully understood, but interactions with the ECS may, to some extent, explain the role of TRPV in this dysregulation [30]. 

The discovery of cannabinoid receptors allowed one to identify its endogenous cannabinoids, such as the endogenous partial agonist anandamide (AEA) and the endogenous full agonist 2-arachidonylglycerol (2-AG) [31], with a greater affinity for CB_1_ and CB_2_ than AEA [32,33], or virodhamine (CB_1_ receptor antagonist and CB_2_ receptor agonist) derived from arachidonic acid and ethanolamine [34].

The abovementioned AEA and 2-AG are the best-known endocannabinoids, although other ECS neurotransmitters, such as 2-arachidonyl glyceryl ether (2-AGE), *N*-arachidonoyl dopamine (NADA), oleamide (cis-9,10-octadecanoamide (ODA)), *N*-arachidonylglycine (NAGLy), palmitoylethanolamide (PEA), stearoylethanolamide (SEA), and oleoylethanolamine (OEA) [33], should also be mentioned, since they all have an affinity for cannabinoid-like G-coupled receptors. The endocannabinoid system is also formed by synthesizing and degrading enzymes, such as AEA-synthesizing enzymes (*N*- acylotransferase (NAT), *N*-acyl phosphatidylethanolamine phospholipase D (NAPE-PLD), and fatty acid amide hydrolase (FAAH)) and 2-AG-regulating enzymes (diacylglycerol lipase (DAGL) and monoacylglycerol lipase (MAGL)) [33,35]. Moreover, it has been recently discovered that glycerophosphodiester phosphodiesterase 3 (GDE3) acts as an ecto-enzyme and converts bioactive lysophosphadylinositol (LPI) to monoacyloglycerols (MG), including 2-AG, and activates CB_1_R as well as CB_2_R signaling in mammalian cultured cells [36,37]. 

CB_1_R is primarily located in the central and peripheral nervous systems, i.e., in the cerebral cortex (neocortex), with a high accumulation in the cingulate cortex, and the frontal and motor cortex. It is also present in the olfactory structures of the brain and the hippocampus, where the accumulation is exceptionally high, as well as in the amygdala, striatum, cortex, deep cerebellar nuclei, brain stem, spinal cord, diencephalon, and hypothalamus, where, in contrast, its accumulation is relatively low [38]. It should be noted that CB_1_R is the most common receptor of GPCRs in the mammalian central nervous system (CNS) [39,40]. Besides, CB_1_R is found in adipocytes and muscles, adrenals, pancreas, liver, gastrointestinal cells, and other tissues [41]. CB_2_R is formerly considered a peripheral receptor due to its location in spleen macrophages. However, cannabinoid receptor type 2 has also been found in some parts of the brain, such as the striatum, hypothalamus, cerebral cortex, hippocampus, amygdala, and substantia nigra [41,42]. Nevertheless, the immune system is the primary location of the CB_2_ receptor. The CB_2_ receptor was found in macrophages, including osteocytes, osteoclasts, Kupffer cells, and B lymphocytes, and in each organ with immune cells, including the cardiovascular system, gastrointestinal tract, and reproductive system, and plays an important role in inflammatory processes (Table 1) [43,44,45,46].

## 2. Role of the Endocannabinoid System in Metabolic Process Regulation 

**Figure 2:**
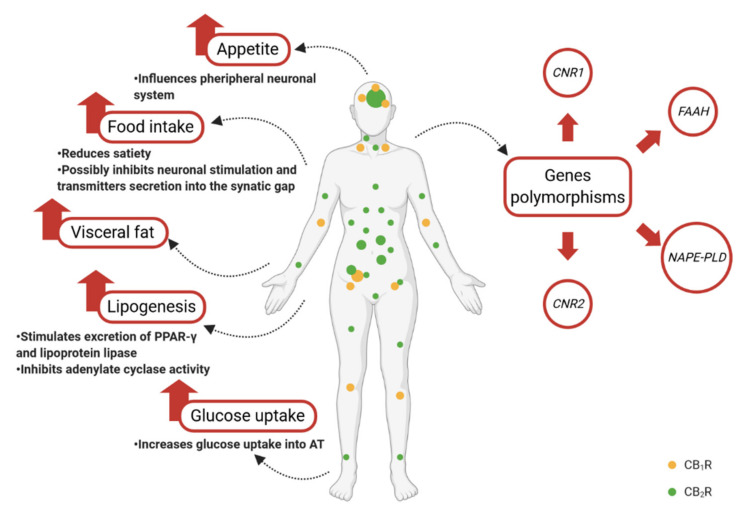
Schematic illustration of the endocannabinoid system role in the regulation of metabolic processes. CB1R—cannabinoid receptor type 1; CB2R—cannabinoid receptor type 2; CNR1—endocannabinoid type 1 receptor gene; CNR2—endocannabinoid type 2 receptor gene; FAAH—fatty acid amide hydrolase; NAPE-PLD—*N*-acyl phosphatidylethanolamine phospholipase D.

Endocannabinoids are involved in the physiological regulation of the body’s homeostasis, stimulating food intake and hunger, as well as shifting energy balance toward energy storage [50], by means of acting on peripheral tissues, such as adipocytes, hepatocytes, islet cells, the gastrointestinal tract, and skeletal muscles (Figure 2). 

### 2.1. Adipose Tissue

The ECS promotes fat storage in adipocytes by intensifying adipogenesis and acts directly by increasing triacylglyceride (TAG) production. It has been shown that the blockage of CB_1_R decreases adipocyte proliferation, while adipocyte differentiation is directly preceded by multiple increases in AEA and 2-AG levels in mice [51,52]. CB_1_R stimulation is accompanied by an increase in peroxisome proliferator-activated receptor gamma (PPAR-γ) receptors, which play an essential role in adipocyte proliferation and increase the size and quantity of TAG in adipocytes of diet-induced obese mice [51,53]. Additionally, CB_1_R activation decreases adiponectin expression and increases leptin expression in mouse white adipose tissue (WAT) [54]. 

### 2.2. Lipogenesis and Lipolysis

CB_1_R activation in mice stimulates the expression of PPAR-γ and lipoprotein lipase, which increases the availability of substrate for TAG production, stored in adipocytes [55]. Moreover, CB1 inhibits adenylate cyclase activity, which inhibits the activity of 5′AMP-activated protein kinase (AMPK), and further of AMPK-associated lipolysis, which reduces fatty acid synthase (FAS) inhibition and lipolysis. CB_1_R stimulation also directly increases FAS expression. On the other hand, CB_1_R increases glucose uptake into the adipose tissue by directly affecting glucose transporter type 4 (GLUT4) in adipocytes. These activities under physiological conditions are regulated by autocrine mechanisms in the adipose tissue. However, under pathological conditions, these actions cause dyslipidemia, i.e., mainly an increase in TAG and low-density lipoprotein (LDL) in the serum, and play a crucial role in developing insulin resistance (IR) [20].

### 2.3. Brown Adipose Tissue

CB_1_R is present in brown adipose tissue (BAT) adipocytes. Previous studies suggest that CB_1_R activation in BAT is based on the inhibition of signals from the sympathetic nervous system (SNS), which decreases thermogenesis [56]. Moreover, peripheral CB_1_R blockade in BAT can provide a new approach to treating obesity and lowering cardiovascular risk. In fact, chronic CB_1_R antagonism has been associated with activation of BAT thermogenesis and weight loss in diet-induced obese mice and rats by both peripheral and CNS-located CB_1_R [56,57]. As Eriksson et al. demonstrated, CB_1_R can be a promising surrogate biomarker for BAT, which would be helpful in further investigation of the activation and regulation of BAT and energy expenditure [58]. Similar results were obtained in the study by Boon et al., where CB_1_R blockade with rimonabant in mice enhanced energy expenditure and reduced dyslipidemia [59]. However, EC signaling via CB_1_R could also provide new approaches to treating obesity and improving metabolism in humans [60].

### 2.4. Liver

As mentioned earlier, CB_1_R is expressed in the liver, promoting the synthesis and storage of TAG [61]. The activation of CB_1_R in wild-type mice resulted in increased expression of sterol regulatory element-binding protein-1c (SREBP-1c) and subsequent expression of related lipogenic enzymes, e.g., FAS and acetyl-CoA carboxylase. Under pathological conditions, it significantly contributes to the development of non-alcoholic steatohepatitis (NASH), further hepatic fibrosis, and dyslipidemia or dyslipoproteinemia [62,63,64]. Moreover, CB_1_R activation causes an expansion of the adipose tissue in the liver, which causes IR [65]. In contrast, the ECS inhibits adiponectin expression, which stimulates liver AMPK and fatty acid entrance into mitochondrial oxidation pathways. It proves that in addition to the direct effect on liver metabolism, the ECS also indirectly decreases the oxidation of fatty acids in the liver in vivo [66]. Furthermore, CB_2_ is also expressed in hepatocytes, and CB_1_R antagonists and CB_2_ agonists protect the liver from toxic failure [67].

### 2.5. Skeletal Muscles

The action effect of CB_1_ in the muscles is associated with the regulation of glucose uptake through the modulation of insulin sensitivity. Under physiological conditions, the activation of CB_1_ in mice results in a decrease in glucose uptake and oxygen consumption by inhibiting fatty acid oxidation [68,69]. 

### 2.6. Pancreas

It has been demonstrated that CB_1_R and CB_2_ are present in alpha and beta islet cells, although their stimulation has a different result. The activation of CB_1_ increases insulin secretion from beta cells and glucagon from alpha cells in mice. CB_2_ stimulation decreases insulin secretion from beta cells and glucagon from alpha cells. These data suggest, therefore, that the glucose plasma concentrations induced by the ECS are not directly associated with pancreatic hormones; however, they are associated to a greater extent with the modulation of peripheral insulin sensitivity dependent upon the ECS [20,70,71].

### 2.7. Gastrointestinal Tract

The ECS reduces the feeling of satiety, which increases the frequency and quantity of food intake. Simultaneously, the ECS decreases gastric juice secretion, intestinal peristalsis, and bowel content passage. This, in turn, results in a higher absorption of nutrients, which could lead to weight gain and further obesity. These processes are influenced by receptors located in the gastrointestinal tract (GT) and the peripheral nervous system [72,73,74]. In fact, a high-fat diet-specific increase of AEA and 2-AG in the jejunum suggests the presence of positive feedback loops. The inhibition of this loop could reduce the consumption of fat-rich products, thus providing a therapeutic solution [75,76]. Furthermore, endocannabinoids are also produced in the gastrointestinal tract, e.g., in the small intestine. On the basis of the mice model, it has been indicated that after 24 h fasting, the level of AEA in the GT increases sevenfold, which can affect the nutritional status, energy balance, lipoprotein metabolism, glucose homeostasis, and even nutritional behavior [39,72].

## 3. Mechanisms of Hunger and Satiety in Obesity

Obesity and its comorbidities, such as coronary heart disease, hypertension, gout, diabetes, or several types of cancers, result from excessive accumulation of adipose tissue, which stems from consuming much more energy than is necessary [77]. However, specific homeostasis helps to maintain proper body weight for a longer period. An increased calorie density of widely available foods can disrupt homeostasis, hence leading to obesity [78]. Interestingly, the balance system is more sensitive to hunger than to satiety, since in the past it was difficult to predict whether and when the next meal will be consumed. Therefore, a tolerance for excessive caloric intake is essential for the development of obesity and metabolic disorders. In fact, appetite is regulated by a system of central and peripheral signals that can modulate the individual reaction to the provided nutrients. A central control of food intake is controlled by the hypothalamus, brain stem, neuropeptide signaling systems, and monoaminergic, and endocannabinoid systems. A peripheral control of food intake involves gastrointestinal satiation signals through hormones like cholecystokinin (CCK), glucagon-like peptide 1 (GLP-1), and neuropeptide Y (NPY). Signals are transmitted to the solitary nucleus (SN) in the brain stem through the vagus nerve. Satiety, associated with a portion size, suppresses hunger and ends food intake. Physiological and psychological mechanisms release signals from different gastrointestinal tract locations, including the stomach, proximal and distal parts of the small intestine, and colon, to the brain [79]. Generally, satiety is defined as a period between meals without the feeling of hunger [80], which is impacted by both long-term signals from body energy storage and short-term signals from the gastrointestinal tract [81]. NPY constitutes an essential neurotransmitter in the brain, increasing during hunger and decreasing during a meal [82]. In the course of weight regain among people with obesity, its role is particularly essential, since body weight reduction reduces leptin concentrations and activates NPY, which is associated with hyperphagia and decreased energy usage. On the other hand, proopiomelanocortin (POMC) has an anorexigenic effect. Therefore, mutations in POMC are associated with increased food consumption and the development of obesity [83].

Endocannabinoids (EC) are anabolic lipid mediators responsible for increased food consumption, energy storage, and lower energy expenditure. They are essential in regulating hormonal and metabolic changes in obesity. For instance, in the mice brain, a selective CB_1_ receptor agonist suppressed food intake [84]. Additionally, the amount of CB_1_in the hypothalamus decreased following exposure to leptin. Thus, the orexigenic effect of CB_1_has been associated with the function of the peripheral neuronal system, regulated by leptin and associated with the development of overweight and obesity [85]. In fact, leptin reduces calcium inflow and further inhibits EBC synthesis, which reduces appetite [86]. Moreover, EC can also modulate energy intake through peripheral mechanisms not associated with appetite. This confirms the resistance to weight gain induced by diet in CB_1_ −/− mice as compared to wild-type mice fed with the same diet [62].

Taste, flavor, or texture stimulates food intake, but they are also essential in achieving the feeling of satiety. As food consumption progresses, signals from the GT (extending stomach wall, ghrelin, peptide hormones) and an increase in glucose levels lead to food consumption termination. However, food palatability stimulates further consumption and can increase a portion intake by 40% in comparison to taste-neutral products [87]. Waiting for a meal or eating time is associated with faster satiety [88]. Furthermore, mental work and sleep deprivation negatively affect satiety and increase the amount of consumed food by means of unstable glycemia and cortisol levels [89]. Moreover, the low*satiety phenotype, i.e., one of the phenotypes of appetite, which favors obesity has been found [90]. In addition, meal composition affects satiety, and according to research data, high-protein meals ensure higher satiety during body weight reduction [91]. In healthy, normal-weight individuals, the consumption of a high-protein diet is associated with higher and longer satiety during the day than the consumption of a diet with a regular amount of protein [92]. In fact, the consumption of protein products with carbohydrates stimulates GLP-1, which increases insulin secretion. Additionally, satiety following high-protein consumption is also associated with increased energy expenditure, which further increases oxygen consumption and body temperature, as well as promoting satiety [93].

## 4. Microbiota, Hunger, and Satiety in Obesity

Intestinal microbiota are a complex population of various microorganisms that can positively affect the human body. However, microbial changes may promote the development of several diseases, in particular metabolic diseases. Microbial changes affect the mechanisms of hunger and satiety, produce neuroactive substances and short-chain fatty acids (SCFA), and thus regulate nutritional behaviors associated with food consumption. *Lactobacillus* and *Bifidobacterium* strains are known to produce gamma-aminobutyric acid (GABA) [94], whereas *L. reuteri* produces histamine, and *L. plantarum* produces acetylcholine [95]. Furthermore, intestinal microorganisms can also moderate intestinal permeability and bile acid metabolism. In fact, studies on mice have shown that probiotic supplementation decreases hunger [96]. The brain–gut axis affects appetite and, therefore, affects the energy state of the host [97,98]. The concentration of catecholamines increases under the influence of β-glucuronidase, produced by bacteria, which can also affect satiety [99]. Additionally, gut bacteria can also modulate serotonin production, which affects gastrointestinal motility and intestinal peptide secretion [99].

A lower response to sweet and greasy flavor was observed in obesity, resulting in increased appetite for these flavors and their higher consumption [100]. Saccharine-sensitive rats have a different intestinal microbiota composition than rats less vulnerable to saccharine [101]. The activation of the immune system causes a decrease in taste receptor cells and taste buds in the tongue [102]. These changes in people with dysbiosis are initiated by the Toll-like receptor and interferon receptor (IFN) types I and II [103], and a similar effect is observed following the influence of lipopolysaccharides (LPS). On the other hand, the administration of LPS to mice results in lower expression of sweet taste receptors and a lower response for saccharose [104]. Moreover, dysbiosis among individuals with obesity increases intestinal permeability, serum lipopolysaccharide levels, and enhances the synthesis of CB_1_R and the expression of macrophages [105]. Furthermore, it increases the inflammatory state, the infiltration of pro-inflammatory macrophages in the adipose tissue, and the accumulation of adipose tissue. Conversely, the antagonists of CB_1_R decrease intestinal permeability and LPS levels [106].

In a study by Maria Isabel Queipo-Ortuñ, higher amounts of Proteobacteria and Bacteroidetes and lower amounts of Actinobacteria and Firmicutes were observed in rats fed a restrictive diet. Additionally, an increase in Lactobacillus and Bifidobacterium was observed in the group with physical activity. In fact, a positive correlation between the amount of Bifidobacterium, Lactobacillus, and leptin and a negative correlation between Clostridium, Bacteroidetes, Prevotella, and leptin were observed. On the other hand, ghrelin was negatively associated with the number of Bifidobacterium and Lactobacillus and positively associated with Bacteroidetes and Prevotella [107]. 

## 5. Cannabinoids/Endocannabinoid Control of Food Intake

ECS activity in the central nervous system—mostly in the limbic system and hypothalamus—is well documented. The endocannabinoid system plays an essential role in connecting gastrointestinal tract activity and the body’s energy economy [50]. The ECS works by increasing both appetite and the motivation to seek food. This mechanism is regulated by nourishment and anorexigenic transmitters produced by the hypothalamus, e.g., corticotropin-releasing hormone (CRH), melanin-concentrating hormone (MCH)**,** and hypocretin [108,109]. 

It is assumed that the ECS operates at the cellular level by inhibiting neuronal stimulation and transmitters secretion into the synaptic gap [110]. The EC level in the hypothalamus is physiologically regulated by hormones reflecting the organism’s metabolic conditions, e.g., leptin, ghrelin, cholecystokinin, and glucocorticosteroids. The use of cannabinoids increases food intake [111]. Studies on rodents have indicated that those with 60% deficiency of CB_1_R in the hypothalamus are less sensitive to a non-nutritive effect of rimonabant (a selective agonist of CB_1_R, previously used as an anti-obesity treatment and then excluded due to multiple adverse reactions, mostly psychiatric). Interestingly, it has been suggested that the leptin effect in the hypothalamus is mostly associated with ECS signaling, since no leptin-induced appetite suppression effect was observed in rodents. Moreover, in another study, ghrelin also reduced this effect [54,112].

It has been suggested that several neuronal connections, directly related to eating behaviors, change their function from stimulation to obesity inhibition. Therefore, EC’s presynaptic inhibiting effect on the expression of transmitters stimulates the expression of pro-nourishing transmitters [54]. Considering that leptin negatively regulates the expression of CB_1_R in the hypothalamus and the fact that leptin resistance is common, leptin resistance impairs negative leptin control of the ECS at the hypothalamus level, causing an increase in subsequent pro-nourishing behaviors [4,85,113]. Moreover, the administration of the reverse CB_1_R agonist restores leptin sensitivity and has an anti-obesity effect in mice [114]. Other rodent studies revealed an increase of 2-AG in the hypothalamus following a high-fat diet (regardless of chronic or acute stimulation). The selective activation of CB_1_R in the central nervous system resulted in resistance to obesity induced by a high-fat diet. Moreover, the inactivation of CB_1_R in the central and sympathetic nervous systems caused an increase of thermogenesis [54,74,85,115,116].

The role of the limbic system in the control of food intake is mainly based on hedonistic fulfilment and needs—in this case, the estimation of taste and related behaviors. The nucleus accumbens, where endocannabinoid and opioid receptors are found, plays the most crucial role in assessing food type. Additionally, the nucleus accumbens has a unique link with the lateral hypothalamus, where the ECS performs a significant function [117,118]. In fact, it has been proven that dopamine activity in the nucleus accumbens is associated with the classification of hedonistic impulses and that CB_1_R blockage in this structure inhibits dopamine expression in response to pleasurable food [117,119]. Furthermore, the vagus nerve contains CB_1_R, CCK, and leptin receptors and is also responsible for maintaining homeostasis. It transfers information from organs to the locus coeruleus concerned with the regulation of digestive processes. CCK is secreted from the duodenum during eating and reduces food intake via the vagus nerve. In addition, leptin negatively regulates CB_1_R levels, also in vagus nerve endings, which suggests another theoretical ECS-related mechanism of reducing food consumption [85].

Moreover, chronic activation of CB_1_R may increase processes associated with hyperlipidemia, diabetes, or cardiovascular events among hedonic patients with obesity [20,120]. Furthermore, several dietary factors, e.g., dietary secondary metabolites, can affect the ECS, and high-calorie and high-fat diets can modulate the CB_1_/CB_2_ ratio and, thus, enhance food intake in several cases [121,122]. EC can also moderate food reward, whereas EC agonists can increase the palatability and hedonic value of food [123]. Interestingly, there is a possible 6-n-propylthiouracil (PROP) taste sensitivity association with the BMI, lipid parameters, and circulating endocannabinoids. Therefore, lower concentrations of AEA or 2-AG in normal-weight nontasters versus normal-weight supertasters can counteract the excess adipose tissue accumulation. In contrast, obesity can disrupt the abovementioned adaptive mechanism, since Carta et al. also noted an opposite correlation between plasma AEA and 2-AG concentrations, as well as the PROP phenotype. As a result, nontasters had 62% higher levels of endocannabinoids than supertasters [124]. It is worth mentioning that the ECS is also dysregulated in eating disorders, and EC dysregulation can be a modulating factor of rewarding binge-eating or self-starvation; however, data regarding this issue remains limited [125]. 

## 6. Genetic Determinants of Obesity in the Context of the Endocannabinoid System

The endocannabinoid system contributes to food control consumption through central and peripheral mechanisms. The ECS is involved in lipid and glucose metabolism control, lipogenesis stimulation, and visceral fat accumulation [126,127,128,129]. Since genetic factors modified by the environmental factors control body weight maintenance, genetic variants affecting the ECS may be essential subjects in the pathophysiology of obesity. 

It is frequently suggested that the main factor contributing to varying degrees of obesity susceptibility may be epigenetics and gene expression [130,131,132]. First studies concerning the role of DNA methylation in obesity were conducted in 2013 by Xu et al., in which the authors identified several CpG sites and DNA methylation variances associated with obesity in young African Americans [133]. Most importantly, it was demonstrated that differential methylation and differential variability can determine the risk of obesity in approximately 70%in humans. Since then, research regarding the role of DNA methylation and histone modification in obesity has been initiated [133,134,135,136,137,138,139].

On the basis of these studies, researchers managed to identify tissues (adipose tissue, blood, skeletal muscle stem cells) and genes (*ABCC3*, *GRB10*, *H19*, *MOGAT1*, *PDGFA*, *PRDM16*, *PRKCE*, *ATP10A*, *IRS1*, *JARID2*, *KCNQ1*, *ABCG1*, *FAM123C*, *FHL2*, *KLF14*, *PHOSPHO1*, *ZNF518B*, *ADCY5*, *CDKN1A*, *FTO*, *INS*, *KCNQ1*, *PDE7B*, *PDX1*, *PPARGC1A*, *SEPT9*, *SOCS2*, *TCF7L2*) associated with obesity and related phenotypes (BMI, waist circumference). The determination of obesity susceptibility genes can contribute to the explanation of weight regulation mechanisms, food intake control, and fat distribution, potentially indicating new approaches to preventing and treating obesity [140]. 

Recent studies have shown that the endocannabinoid system can also be epigenetically modulated by drugs, alcohol, and diet. These modulations mainly include the *CNR1* gene but also the hydrolyzing enzyme FAAH. The epigenetic modulations mechanisms concern global and gene-specific DNA methylation changes, histone acetylation and deacetylation, and the production of specific miRNAs [141,142,143,144,145,146].

It has been demonstrated that peripheral ECS activity is epigenetically modulated by diet. It turns out that the compound affecting the epigenetic modulation of CNR1 expression in vivo and in vitro is the commonly used extra virgin olive oil (EVOO), as evidenced by the ~50% reduction in CpG methylation status of the *CNR1* promoter and expression of CB_1_R modulators, i.e., miR23A and mir-301a (involved in the pathogenesis of colorectal cancer) in rats exposed to short- and long-term dietary EVOO. Moreover, Francesco et al. showed that EVOO and its phenolic components were able to selectively regulate CNR1 gene expression in Caco-2 cells due to the hypermethylation of the *CNR1* promoter. Interestingly, the frequency of CNR1 methylation is also quite high in human colorectal cancer and reaches about 77% and seems to be a relevant mechanism for cancer progression [147]. A study by Pucci et al. revealed a significant and selective increase in *CNR1* gene expression in high-fat-diet rats at the beginning of obesity development and after 21 weeks of high dietary exposure, with a simultaneous selective and significant decrease in DNA methylation at specific CpG sites in both gene promoters in overweight rats [148]. In fact, CB_1_R is present in human subcutaneous adipocytes, encoded by the *CNR1* gene, alterations in which, related to obesity traits, are frequently described in the literature. It is well established that CB_1_R activation leads to an increase in energy storage, which occurs via an increased motivation to consume food and to decreased satiety [149]. Increased levels of CB_1_R and endocannabinoids are observed during adipocyte differentiation. According to Ravinet Trillou et al., *CNR1*-deficient mice were lean and resilient to diet-induced obesity [150]. The dysregulation of the endocannabinoid system seems to play a crucial role in human obesity [149], which can be demonstrated by significant abdominal fat accumulation [151], as well as the fact that *CNR1* gene variants are associated with an increased appetite, BMI, waist circumference, and skin-fold thickness, and even the appearance of metabolic syndrome [152,153]. The important role of the ECS in the pathophysiology of obesity can be proven by the fact that a blockade of *CNR1* by means of rimonabant leads to a significant reduction in food intake and weight loss [154,155]. Moreover, these findings indicate that *CNR1* gene antagonists improve glucose and lipid homeostasis, which occurs independently of weight loss, implying that *CNR1* gene variability may contribute to obesity-related metabolic disorders in view of human obesity.

Benzinou et al. studied 26 single-nucleotide polymorphisms of *CNR1*, in which 12 showed nominal evidence of association with childhood obesity, class I and class II, and class III adult obesity. The study was conducted on 5750 patients and demonstrated that *CNR1* gene variants increases the risk of obesity and modulates the BMI in the European population [152]. The impact of *CNR1* genetic diversity on the BMI was also confirmed by other researches, who demonstrated that the rs1049353 mutant allele is associated with a lower BMI in European populations [156,157,158]. Moreover, it was found that in Caucasians suffering from anorexia or bulimia, the T allele is much more frequent in both homo- and heterozygous individuals than in healthy individuals [159]. Interestingly, homozygotes with the rs1049353-mutated allele correlated with a higher WHR and waist circumference (WC) in obese men and were associated with an increase in childhood obesity [160]. Consequently, it may suggest that CNR1-specific variants may constitute key elements in the understanding of the CB_1_R impact on feeding, fat accumulation, and susceptibility to increased adiposity. A previous review paper [143] regarding the role of ECS genetic polymorphisms in relation to obesity and diabetes was already published in 2019 by Doris et al. On the basis of the data from their review, we attempted to present an up-to-date table describing the associations between ECS obesity-related genes’ single-nucleotide polymorphism (Table 2) However, it is important to bear in mind research reports, such as Muller’s group, investigating eight polymorphic sites of the CNR1 gene (rs9353527, rs754387, rs6454676, rs806379, rs1535255, rs2023239, rs806370 and rs1049353), which do not confirm any association of the variants studied with regard to obesity in children and adolescents [161].

The fact that the endocannabinoid system is closely related to the control of metabolism and energy balance has already been well documented. Due to the pressure in the brain, the CB1 receptor has been mainly considered as controlling glucose and lipid metabolism. Although its level of expression in peripheral cells was deficient, it appeared to influence increasing obesity. It can also be evidenced by the fact that the genetic ablation of *CNR1* leads to weight loss, as well as selective blocking of the CB_1_ receptor, which leads to reduced food intake and body weight loss. Nevertheless, following the CB_2_ discovery as a peripheral receptor and its identification in different brain regions, including the hippocampus, the approach was changed [42,180,181]. *CNR2* was rarely examined in view of obesity, since its locations in the liver, adipose tissue, and pancreatic islet cells have only recently been defined [4]. Romero-Zerbo et al. showed that the overexpression of cerebral CB_2_R affects body weight modulation, leading to a lean phenotype in mice [182]. Additionally, CB_2_ agonists can reduce food intake in lean mice, simultaneously improving weight gain and obesity-related inflammation in diet-induced obese mice [183]. It turns out that the frequently studied variant CB2, Q63R, which affects the reduction in CB_2_R function, is also associated with human nutrition disorders, eating behavior, and energy homeostasis.

Moreover, its genetic ablation leads to adiposity development [184]. Rossi et al. analyzed the effect of rs35761398 (Q63R) on CB_2_Ron obesity modulation in Italian children and adolescents. They found that the less functional missense variant R63 was significantly associated with a high *z*-score body mass index. Additionally, they showed that the CB_2_R reverse agonist AM630 increased inflammatory adipokine release and fat storage, whereas the JWH-133 agonist reversed all effects related to obesity [185]. In turn, Ketterer et al. investigated five tagging SNPs of the *CNR2* locus (rs2229579, rs3123554, rs9424398, rs4625225, rs2501392) concerning the association with the BMI, weight, total body fat as a measure for adiposity, and the WHR as an estimation of body fat distribution. They observed a significant association of rs3123554 with weight (*P* additive inheritance model = 0.0062), as well as a lower BMI and body fat. Interestingly, the association with the BMI was limited to females, which may constitute evidence of the identified interactions between the ECS and sex hormones [186]. Other studies have also highlighted the role of the rs3123554 minor allele in the body weight, insulin and leptin higher levels, triglycerides, and homeostatic model assessment—insulin resistance (HOMA-IR) based on the example of the Spanish cohort [187]. Summarizing all the reports described above, we can conclude that CB_2_R may constitute a new pharmacological target for the treatment of obesity in the future. However, as the role of CB_2_ is still not fully understood, it requires intensive research. 

Analyzing genes encoding enzymes involved in the synthesis or the degradation of endocannabinoids (FAAH, MAGL, DAGL, and NAPE-PLD), we found that any available studies described the association of MAGL and DAGL polymorphism and their role in the incidence of obesity, whereas FAAH was defined as an obesity-related factor. Sipe et al. showed that a naturally occurring missense polymorphism rs324420 (c.C385A) and the A/A genotype were associated with overweight and obesity in 2667 white (*P* = 0.005) and black (*P* = 0.05) subjects but not in Asians [174]. Yagin’s findings confirmed these results and demonstrated that the prevalence of the c.385A allele was more frequent in overweight/obese individuals, and the changes in the FAAH gene were associated with higher anthropometric indices, as well as the A/A genotype, which significantly increased the risk of obesity in Iranian women [176]. Moreover, the association of rs324420 with an increased risk of obesity and higher triglyceride levels was demonstrated in different European cohorts [175,187,188,189,190]. Additionally, Balsevich et al. demonstrated that an endocannabinoid-dependent signaling mechanism contributes to the hyperphagic actions of leptin, which increases FAAH activity and reduces AEA signaling in hypothalamic regions, leading to a reduced food demand. This mechanism is also modulated by the genetic variant C385A of the FAAH gene. It follows, therefore, that reducing leptin susceptibility to leptin in individuals with a mutated 385A allele may lead to an increased risk of developing obesity with the related metabolic complications [191].

On the other hand, we found reports that presented contradictory results, suggesting that FAAH gene polymorphism and mutant allele carrying were not related to overweight or obesity. Jansen et al. found no correlation between FAAH 385A allele Danish subjects and the BMI, WHR, WC, and HOMA-IR [192], as well as Papazoglou et al., who did not confirm the link to severe obesity with or without the diagnosis of metabolic syndrome in Greeks [193]. Nevertheless, rs324420 was associated with elevated anandamide levels in the Brazilian population, which may prove to support the cannabinoid antagonist treatment strategies in overweight-related disorders [194].

NAPE-PLD encodes one of the enzymes of endocannabinoid synthesis and participates in the production of anandamide, a CNR1 agonist. NAPE-PLD and other ECS components are the subject of research in terms of drug targets in the treatment of several diseases, including obesity and metabolic comorbidities. Wangensteen et al. showed strong evidence that a common haplotype in NAPE-PLD (rs13232194, rs17605251, rs11487077, rs12540583, rs6465903) was associated with severe obesity. Additionally, they observed that one SNP, rs17605251, in NAPE-PLD was nominally associated with BMI in Norwegians [195].

## 7. Summary and Conclusions

The importance of the endocannabinoid system in regulating metabolic pathways in the human body is increasingly becoming the subject of research. The discovery of CB1 and CB2 receptors, their agonists, and their antagonists has enabled research on the potential role of the EC system in various physiological and pathological processes, such as appetite regulation, energy balance, food intake, fat deposition, hepatic lipogenesis, and glucose homeostasis. This indicates that the ECS can be overactive in obese patients, thus promoting metabolic processes resulting in weight gain, lipogenesis, insulin resistance, and dyslipidemia. Therefore, innovative new obesity treatments are highly desirable, and blocking the endocannabinoid system seems to be a crucial solution. Studies demonstrated that selective CB_1_R agonists can be used for the pharmacological treatment of obesity. Rimonabant was introduced in the European Union in 2006 and was tested in three randomized clinical trials. The study was entitled Rimonabant in Obesity (RIO), in which its influence on body weight reduction among 5588 overweight (BMI > 27 $\frac{\mathrm{kg}}{m^{2}}$) and obese patients with comorbidities (dyslipidemia and hypertension) was assessed [196]. A significant weight reduction, an improvement in the serum lipid profile (RIO-lipids), an increase in HDL cholesterol, and a decrease in TG were observed [197]. Furthermore, in the RIO-Diab clinical trial, an improvement in insulin sensitivity and glycemic control and a decrease in fasting glucose levels, the HOMA-IR index, and glycated hemoglobin levels were also demonstrated [198]. Although RIO trials proved the safety of rimonabant, the approval was rejected, since it induced symptoms of depression as well as anxiety and resulted in other side effects (nausea, emesis, diarrhea, vertigo, and headaches). The negative influence of rimonabant on the gastrointestinal tract is associated with the blockade of CB_1_R [199]. At this point, it is worth mentioning that individuals with obesity have an increased risk of the more severe course of severe acute respiratory syndrome coronavirus 2 (SARS-CoV-2) infection [200,201]. The use of rimonabant in order to decrease inflammation, a vital hallmark of obesity and SARS-CoV-2 infection, could provide a potential beneficial treatment in terms of a severe COVID-19 course [202]. Therefore, rimonabant use should be considered in clinical trials for patients with SARS-CoV-2 infection [203]. Although rimonabant was withdrawn from the market, attempts on testing other CB_1_R second-generation antagonists were made. URB447, i.e., a CB_1_R and CB_2_R antagonist, decreased the intake of food and body weight in mice (also peripherally) and was similar to rimonabant with regard to the polar surface and other physicochemical properties [204]. AM6545 was referred to as the neutral antagonist due its limited penetration to the brain [205], and its administration increased leptin sensitivity and the metabolic profile in obese mice, although it presented limited oral bioavailability. BPR697, reported by studies in the Health Research Institutes (NHRI) in Taiwan, exhibits a low brain-to-plasma concentration ratio (B/P = 1/23), similar to rimonabant [206]. BPR697 induced weight loss in diet-induced obese mice, without altered food intake, and decreased hepatic lipid accumulation. *N*-cyclohexyl-4-[1-(2,4-dichlorophenyl)-1-(p-Ttolyl)methyl]piperazine-1-carbo amide exhibited a lower B/P ratio than rimonabant, although it presented a similar effect on weight loss in diet-induced obese mice [207]. AJ5012 and AJ5018 were created due to a structural change in rimonabant. Nevertheless, although their B/P ratio was significantly lower than rimonabant, their ability to decrease adipose tissue inflammation was similar to rimonabant. However, their oral bioavailability remains to be investigated [208]. According to Matthews et al., a peripherally selective tetrahydroindazole derivative (2p compound), used in diet-induced obese mice, showed a beneficial effect on plasma glucose [209]. Zhang et al. discovered a new class of 6-benzhydryl-4-amino-quinolin-2-ones [210]. The 6a compound presented a long half-life following oral and intravenous administration; however, due to high affinity to rimonabant, it presented time-dependent brain accumulation. Nevertheless, this observation could be understated due to the use of non-perfused brains. The administration of the 6a compound resulted in weight loss and increased insulin sensitivity, even when lower doses were administered. These results are promising, although the neuropsychiatric safety of these compounds should be investigated further.

Taranabant is a CB_1_R inverse agonist the effectiveness of which was proven in preclinical trials—it resulted in effective weight loss in diet-induced obese rats when 30% of CB_1_R was occupied [211]. Phase III clinical trials on humans were stopped in 2008 due to the high level of CNS side effects, mainly depression and anxiety [212,213]. In recent years, many CB_1_R-selective drugs have been withdrawn at different phases of clinical trials. Drinabant, a highly selective CB_1_R agonist, presented anti-depression and food-limiting effects in animal models and reached phase IIb clinical trials, but it was shortly withdrawn since it induced symptoms of severe psychiatric disorders in humans [211,214,215]. Failures of clinical trials limited the studies on humans, and currently, CB_1_R agonist are usually tested in laboratory conditions. 

In the study by Radiszewska et al. on Wistar rats, a stimulation of peripheral CB_1_R by its agonist (WIN 55,212-2) had an anorexigenic effect and induced activity of exendin-4, a peptide agonist of the glucagon-like-peptide 1 [216]. It suggests a double activity of the ECS and could be used in the pharmacotherapy of obesity. 

Studies conducted by Palomba et al. indicated that the activation of CB_1_R resulted in overexpression of PPAR-γ in adipocytes, thus suggesting a potential role of thiazolidinediones in this mechanism [51,217]. 

Taking into consideration the undeniable role of CB_1_R in the development of obesity and the role of CB_1_R agonists in obesity treatment, it is essential to further develop the research on CB_1_R-active substances, which have great potential in the treatment of obesity and obesity-related comorbidities. 

It has been proven that exogenous factors are not the only elements responsible for the dysregulation of nutritional homeostasis. Our review presented evidence that there is a link between ECS gene polymorphisms and the risk of obesity. Research on ECS dysfunction could provide more precise knowledge of both the pathogenesis and the mechanisms of obesity, as well as provide new therapeutic programs. Previously introduced ECS-related therapeutic programs have been withdrawn due to mental disorders they resulted in; however, the endocannabinoid system remains the subject of numerous research studies.

## Figures and Tables

**Table 1:** Presence of CB_1_R and CB_2_R in human tissues [47,48,49].

Concentration Tissue	CB_1_R	CB_2_R
Adrenals	+ +	0
Appendix	+ +	+ +
Bone marrow	Low	+
Brain	+ + + +	0
Colon	+	Low
Duodenum	+	Low
Endometrium	+	Very low
Esophagus	+	Very low
Fat	+ + +	0
Gall bladder	+	
Heart	Low	0
Kidney	Very low	0
Liver	0	Very low
Lung	+ +	Low
Lymph node	+ +	+ + +
Ovary	+	0
Pancreas	Very low	0
Placenta	+ +	Very low
Prostate gland	+	Low
Salivary gland	+	Very low
Skin	+	Very low
Small intestine	+	Low
Spleen	+	+ + +
Stomach	+	+
Testis	+	Low
Thyroid	+	Low
Urinary bladder	+	+

RNA-sequencing. RPKM, reads per kilobase million. 0, absent; very low, concentrations equal and below 0.03 RPKM; low, concentrations in the range of 0.119–0.031 RPKM; +, concentrations in the range of 0.582–0.200 RPKM; + +, concentrations in the range of 1.272–1.089 RPKM; + + +, concentrations in the range of 3.665–2.735 RPKM; + + + +, concentration equal to 6.155 RPKM; CB1R—cannabinoid receptor type 1; CB2R—cannabinoid receptor type 2.

**Table 2:** Associations found between a single-nucleotide polymorphism in ECS obesity-related genes.

Gene	Polymorphism	Nucleotide Change	Amino Acid Change	Association	Ref
CNR1	rs1049353 (exonic)	c.1359G>A	p.Thr453=	Associated with a specific macronutrient’s intake, low-cholesterol and fat-saturated intakes in Caucasian females	[162]
				Associated with higher fat but not with a metabolic syndrome in postmenopausal Polish women	[163]
				Associated with an increased waist-to-hip ratio and waist circumference in obese Caucasian men	[164]
				Associated with a greater weight loss and a decrease in the BMI	[165]
				Associated with childhood obesity	[166]
				Associated with obesity risk and BMI modulation	[152]
				Associated with a lower BMI	[156]
				Associated with BMI modulation and body weight	[167]
				Associated with visceral and intermuscular fat mass	[158]
				Associated with a lower BMI and fat mass	[168]
				Associated with lower insulin levels	
CNR1	rs806378 (intronic)	c.-63-4495G>A	-	Associated with antipsychotic-induced weight gain in schizophrenia patients	[169]
				Associated with altered gastric functions or satiation	[170]
CNR1	rs806381 (intronic)	c.-64+9621T>C	-	Associated with obesity-related phenotypes in Polish postmenopausal women.	[163]
				Associated with a visceral fat mass	[158]
				Associated with childhood obesity in the French cohort	[152]
				Associated with an increased BMI in the adult Swiss cohort	
				Associated with metabolic effects	[171]
CNR1	rs806368 (3’UTR)	c.*3475A>G	-	Associated with obesity in Japanese men	[172]
				Associated with an increased BMI and waist circumference	[168]
				Associated with triglyceride levels	[173]
CNR1	rs806370 (intronic)	c.-63-1275G>A	-	Associated with circulating levels of HDL-C	
CNR1	rs806369 (intronic)	c.-63-1122A>G	-	Associated with triglyceride levels as well as the total cholesterol level	
FAAH	rs324420 (missense)	c.385C>A	p.Pro129Thr	Associated with increased obesity	[168]
				Associated with a risk factor in overweight/obesity in whites, blacks, and Asians	[174]
				Associated with overweight/obesity but not with binge-eating disorder in Caucasian females	[175]
				Associated with overweight/obesity in Iranian individuals	[176]
				Associated with an increased BMI	[165]
				Associated with larger improvements in glucose, total cholesterol, low-density lipoprotein cholesterol, body mass, and waist circumference in Spanish individuals	[177]
				Associated with insulin improvement and HOMA-R levels with a high-polyunsaturated-fat hypocaloric diet following weight loss	[178]
CNR2	rs35761398 (missense)	c.188_189delAAinsGG	p.Gln63Arg	Associated with childhood obesity and the age of menarche in Italian obese girls	[179]

## Data Availability

Data are available in a publicly accessible. The data presented in this study are openly available in Medline and PubMed databases and on the publisher’s website. The keywords that were used: endocannabinoid system; ECS, obesity; cannabinoid receptors; obesity pathogenesis, and epigenetics of obesity. All data in the text are quoted and all works used are listed in the bibliography along with doi and reference numbers.
